# Predicting Inactivation of *Bacillus subtilis* Spores Exposed to Broadband and Solar Ultraviolet Light

**DOI:** 10.1089/ees.2018.0404

**Published:** 2019-06-04

**Authors:** F.A. Handler

**Affiliations:** Panasynoptics Corporation, Mclean, Virginia.

**Keywords:** airborne pathogens, UV disinfection, UV dose–response, UV inactivation, waterborne pathogens

## Abstract

This study develops general predictive models for the ultraviolet (UV) radiation dose–response behavior of *Bacillus subtilis* spores to solar UV irradiation that occurs in the environment and broadband UV irradiation used in water disinfection systems. The approach is demonstrated using previously obtained experimental survival rates for *B. subtilis* spores deposited on dry surfaces as well as in water and exposed to both narrow band UV radiation as well as broadband UV irradiation from solar exposure and disinfectant lamps. Results are modeled to derive predicted survival rates for spores as a function of irradiance intensity and wavelength, capability for repair, and depletion of available sites for UV damage. The essential features of the approach are expression of the inactivation action spectrum in terms of the probability of an incident photon being absorbed and forming a dimer lesion, and expression of the spore survival as a cumulative binomial distribution for damage. The results provide increased accuracy in estimating dispersed biological hazards, and evaluating the effectiveness of UV air and water disinfectant systems. In addition, the approach for the first time explains the observed reduced inactivation rate in a repair-capable strain compared with a sensitive, repair-deficient strain by accounting for the depletion of available lesion-forming sites due to increasing DNA damage.

## Introduction

The objective of this study is to demonstrate a mechanistic, predictive modeling approach that can accurately predict inactivation of both sensitive strain and wild-type, repair-capable bacillus spores by solar ultraviolet (UV) irradiation (from ∼290 to 400 nm), as well as by broadband UV irradiation (from ∼200 to 400 nm) from disinfection lamps, based on measurements of inactivation of such spores under similar conditions by narrow band UV irradiation at wavelengths spanning the UV range of interest. The approach is designed to explicitly account for the accumulation of inactivating DNA damage (pyrimidine dimers) from exposure to multiple wavelengths of UV and the spore's capability to repair damage from all wavelengths during germination.

The intended application of these results is improved accuracy in predicting inactivation of *Bacillus subtilis* spores in air and in water by solar irradiation and broadband disinfectant lamps, since *B. subtilis* is used as a biodosimeter and an indicator of water disinfection system effectiveness, as well as to be able to apply the approach to other spores such as *Bacillus anthracis* for environmental hazard assessments and *Bacillus thuringiensis* for assessment of biopesticide degradation. Further, the approach is intended to provide insight into the mechanism by which repair-capable strains display markedly lower decay rates (distinguished from a delayed onset of damage) than sensitive strains differing only by lacking repair capability.

The essential feature of the approach is expression of inactivation probability in terms of the per-photon probability of incident photons being absorbed and inducing damage, and calculating spore survival as a cumulative binomial probability distribution for DNA damage, in contrast to traditional approaches expressing inactivation probability in terms of incident fluence. Notation and symbols used are provided in the Supplementary Data.

Predicting inactivation of *B. subtilis* spores from exposure to solar and broadband disinfection lamp UV irradiance is useful for several reasons. The presence of pathogenic microbial organisms in the environment, either indoors or outdoors, in air or in water, can adversely affect humans and livestock. Exposure to UV, either to solar UV, during outdoor transport and dispersion (Handler and Edmonds, 2015), or to solar or low-pressure lamp disinfection systems (McGuigan *et al.*, 2012), or to UV-C (253.7 nm) in UV germicidal irradiation systems (Kowalski, 2009), has been demonstrated to reduce the potential adverse effects by inducing damage to DNA that renders harmful microbial organisms inactive. A wide variety of organisms are susceptible to UV inactivation, including bacterial spores and vegetative cells, viruses and fungi (Kowalski, 2009).

Bacterial spore-forming organisms are of interest because some are pathogenic and, since they are relatively impervious to environmental degradation other than UV inactivation (e.g., desiccation), they are often used to isolate and study UV inactivation phenomena and to serve as biodosimeters of inactivating solar UV radiation (Munakata *et al.*, 2000; Marshall *et al.*, 2011). *B. subtilis* is a Gram-positive spore-forming bacterium, and has been studied extensively as a biodosimeter and as a surrogate for *B. anthracis* (Nicholson and Galeano, 2003).

## Materials and Methods

### Experimental protocols

This work relies on *B. subtilis* exposure data published by Munakata *et al.* (1991, 1996), denoted M91, M96, or M9196 for both papers, and Cabaj *et al.* (2002), which will be denoted C2002.

M9196 experimental protocols are extensively discussed in those references and are only summarized here. M19196 obtained five strains of *B. subtilis*, including a sensitive strain TKJ6321 with excised genes (*uvrA10*, *sp-1*, *polA151*, *hisH101*, and *metB101*), which was deficient in repair of UV-induced spore photoproducts (SPs), and a wild-type strain HA101 with full SP repair capability. In M91, spores were deposited on glass plates and air dried before exposure to narrow band irradiances ranging from 50 to 300 nm derived from a monochromator operating off an electron storage ring. Exposures from 150 to 300 nm were done in a moderate vacuum (10^–3^ Pa). Spores were recovered and plated to produce survival curves for each of the spore strains, and fitted to a simple exponential.

M96 exposures were obtained for the sensitive strain, prepared similarly, but deposited on membrane filters and exposed to nine narrow band sources at wavelengths from 254 to 400 nm, in ambient air, derived from a spectrograph operating off a xenon lamp and grating monochromator. In addition, M96 reported measurements of exposure of the sensitive strain to solar irradiation at the Tsukuba Aerological Observatory for which concurrent spectral irradiance measurements were provided by a Brewster spectrophotometer.

C2002 measured survival of a wild-type, repair-capable *B. subtilis* strain (ATC6633) cultured with previously developed methods (Sommer and Cabaj, 1993). Spores were suspended in water, continuously stirred, and exposed to UV radiation in six different wavelengths from 214 to 352 nm with bandwidths of 20 nm from a 400 W xenon lamp coupled to a single monochromator. Spectral irradiance was measured at the surface of the suspensions with a calibrated spectroradiometer, before and after exposures. Exposures of suspensions to broadband UV used a Philips HPK 125 W lamp filtered with a SCHOTT WG280 cutoff filter and also unfiltered. Incident spectra were measured using the spectroradiometer and reported in C2002.

### Modeling approaches

Mechanisms of UV inactivation of spores have been investigated extensively (Riesenman and Nicholson, 2000; Sinha and Hader, 2002; Weber, 2005; Pattison and Davies, 2006; Setlow, 2006; Kowalski, 2009; Moeller *et al.*, 2009), and involve species-, strain-, and preparation-dependent (1) spore-protective mechanisms, (2) photochemistry of the UV interactions with the DNA, and (3) repair mechanisms available to the spores, typically during germination. A variety of photochemical reactions leading to inactivation involve UV photon-induced excitation of DNA base pairs and formation of a variety of photoproducts that result in lesions in the DNA. In most organisms, UV exposure at wavelengths typical of solar irradiance (from ∼290 to 400 nm) results in a variety of damage products such as cyclobutane pyrimidine dimers (TT-CPD), pyrimidine pyrimidone photoproducts, and strand breaks, but the unique biochemical composition of spores, particularly the presence of small acid soluble proteins (SASPs) bound to the DNA and dipicolinic acid (DPA), results predominantly in the formation of a particular product, a thymidyl–thymidine adduct termed SP (Douki *et al.*, 2005; Setlow, 2006).

Exposure conditions can significantly affect the rate of UV inactivation. Aggregation or clumping of organisms can shield some fraction of the spores from UV radiation, resulting in a lower apparent inactivation rate (Pennell *et al.*, 2008; Kesavan *et al.*, 2014). The water content of spores resulting from dry air exposures, water suspension exposures, or variations in relative humidity during exposure significantly affects the observed inactivation rates [see, e.g., the rates given in Kowalski (2009) under various hydration conditions] (Setlow, 2006; Moeller *et al.*, 2009; Lim and Blatchely, 2012). Photoreactivation has been observed to be absent in *B. anthracis* (Knudson, 1986; Venieri *et al.*, 2013) and only slightly present in *B. subtilis* (Nicholson, 1995).

Nevertheless, the dose response of specific strains of *B. subtilis* spores, when consistently cultured and prepared and exposed to narrow band UV under controlled environmental conditions to well-characterized UV fluence, displays a characteristic dependence, typically exponentially decreasing with increasing UV fluence, possibly with an initial shoulder. The dose response obtained in M91 for narrow band exposures of the *B. subtilis-*sensitive strain displays a simple exponential decay characterized by a decay constant *k_s_*, such that the surviving fraction, *S*, was modeled in M9196 as $S \left( F \right) = A{e^{ - {k_s}F}}$, where *F* is the total narrow band UV energy per unit area delivered, for example, J/m^2^, and *A* and *k_s_* were obtained by regression from the experimental data. The constant *A* for simple exponential decay should be 1, corresponding to complete survival with no exposure, and M91 concluded that since the values obtained for *A* by regression at all wavelengths were within 1 standard deviation of 1, that the variations of *A* were not significant and the decay was well represented by a simple exponential characterized at each wavelength only by the decay constant *k_s_* with units area per unit energy, for example, m^2^/J.

Wild-type strains can repair some amount of SPs (as well as other potential damage) and exhibit a region at low fluence, called the shoulder, in which the inactivation increases less rapidly with fluence than at higher fluence. At higher fluence the decay of a wild-type, resistant strain exhibits an exponential dependence on fluence, characterized by a decay constant *k*_wt_. Such shouldered behavior has typically been modeled by a multitarget model, where $S \left( F \right) = 1 - \;{ \left( {1 - {e^{ - {k_{{ \rm{wt}}}}F}}} \right) ^n}$, in which *n* is called the multitarget exponent and, while typically not constrained to be an integer through regression on data, corresponds to increasing repair capability for increasing values of *n*. Other alternative forms incorporating multiple repair capability have been used, such as a series-event model, $S \left( F \right) = { \frac { N \left( F \right) }  { { N_0 } } } = \left( { { e^ { - { k_1 } F } } \mathop \sum \limits_ { i = 0 } ^ { { n_1 } - 1 } { \frac { { { \left( { { k_1 } F } \right) } ^i } }  { i! } } } \right)$, which expresses *S* as a sum of *n* exponential terms and restricts *n* to be an integer.

The value of the decay constants obtained using a multitarget versus series event can differ by as much as a factor of 2 or 3 [e.g., Ke *et al.* (2009) report a multitarget-derived *k* = 0.15 cm^2^/mJ, and Lim and Blatchley (2009) report a series-event-derived *k* = 0.53 cm^2^/mJ for the same strain *B. subtilis* (ATC6633) in air at the same humidity exposed to narrow band ultraviolet-c (UVC) [254 nm]). For *B. subtilis*, the sensitive, repair-deficient strain exponential decay in narrow band exposures ranges from ∼3 to 20 times faster than the exponential decay region of the repair-capable strain, depending on wavelength (M91). A photochemical basis has not been proposed for the difference in exponential decay rates between sensitive strain and repair-capable strain.

**FIG. 1.**
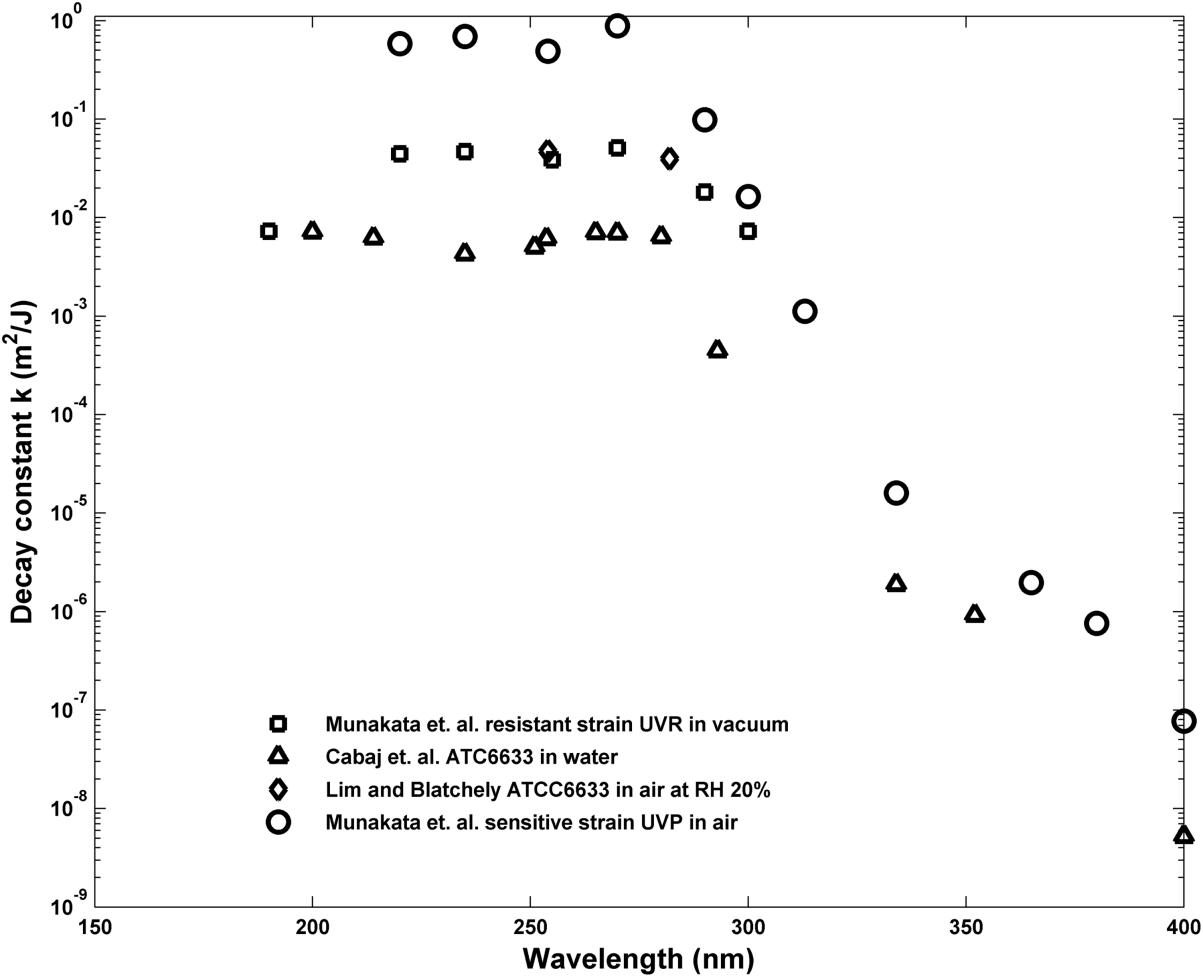
Decay constants for repair-deficient and repair-capable strains of *Bacillus subtilis*. *Circles* plot the narrow band UV decay constants reported by Munakata *et al.* (1991, 1996) for the repair-deficient strain (TKJ 6321, denoted UVP by Munakata *et al.*) deposited and exposed on a surface as a function of wavelength. *Squares* plot a repair-capable strain of *B. subtilis* (HA101, denoted UVR) from Munakata *et al.* (1991) deposited on and exposed on a surface. *Triangles* plot *B. subtilis* strain ATC6633 exposed in aqueous solution reported by Cabaj *et al.* (2002). UVP, ultraviolet polymerase deficient; UVR, ultraviolet resistant.

Inactivation of spores exposed to broadband solar UV as found at the Earth's surface as well as broadband lamp-produced UV is characterized by an inactivation action spectrum (IAS), which is comprised of the decay constants for the spores measured in a set of narrow band exposures spanning the wavelengths found in solar UV at the Earth's surface, from ∼290 to 400 nm, and in water disinfection lamps from ∼220 to 400 nm. Data reported for several strains of *B. subtilis* exposed in air, vacuum, and water are shown in Fig. 1. M9196 reported the decay constants of a sensitive, repair-deficient strain of *B. subtilis*, TKJ6321, denoted ultraviolet polymerase deficient in that reference and exposed to narrow band UV at wavelengths from <200 up to 400 nm in vacuum (M91) and air (M96). M91 also reported the decay constants for a repair-capable strain of *B. subtilis*, HA101, denoted ultraviolet resistant (UVR) in that reference, exposed in vacuum for similar wavelengths up to 300 nm.

C2002 reported decay constants for *B. subtilis* strain ATC6633 (repair capable, wild-type) exposed in aqueous suspension at wavelengths from 200 up to 400 nm. In addition to the decay constant variation with wavelength, several workers (C2002; Wang *et al.*, 2009) have reported that the regression-derived multitarget shoulder parameters, *n*, vary as a function of wavelength. A mechanistic method of relating and accumulating damage over wavelengths has not been proposed. C2002 proposed to use a polychromatic multitarget expression of the form

\begin{align*}
P \left( \cal F \right) = 1 - { \left( {1 - {e^{ - \cal K\cal
F_0}}} \right) ^{ \cal N}} , \tag{1}
\end{align*}

where $P \left( \cal F \right)$ is the survival probability given total polychromatic fluence

\begin{align*}
{ \cal F_0} = \mathop \smallint \limits_{200{ \rm{nm}}}^{400{ \rm{nm}}} F \left( \lambda \right) d \lambda , \tag{2}
\end{align*}

where $F \left( \lambda \right)$ is the fluence per unit wavelength, and the average polychromatic decay constant $\kappa$ is obtained by weighting the wavelength-dependent decay constant *k_w_*( λ) by the wavelength-dependent fluence:

\begin{align*}
\cal K = \frac { 1 }  { { { { \cal F } _0 } } } \mathop \smallint \limits_ { 200 { \rm { nm } } } ^ { 400 { \rm { nm } } } { k_w } \left( \lambda \right) F \left( \lambda \right) d \lambda . \tag { 3 } 
\end{align*}

The average polychromatic multitarget shoulder parameter ${ \cal N}$ was obtained by weighting the wavelength-dependent shoulder parameters *n*(λ) by the wavelength-dependent contribution to inactivation through ${k_w} \left( \lambda \right) F \left( \lambda \right)$:

\begin{align*}
\cal N = {1 \over {\cal K\cal F_0}} \mathop \smallint
\limits_{200{ \rm{nm}}}^{400{ \rm{nm}}} n \left( \lambda \right)
{k_w} \left( \lambda \right)  \;\cal F \left( \lambda \right) d
\lambda . \tag{4}
\end{align*}

Comparing survival predicted by Equation (1) with measurements, C2002 reported differences ranging over more than an order of magnitude. Similarities have been noted between the wavelength-dependent decay constants and the UV absorbance of DNA (C2002; Mamane-Gravetz *et al.*, 2005), but a detailed functional prediction has not been proposed.

### Modeling repair-deficient *B. subtilis* inactivation

The probability of repair-deficient *B. subtilis* survival for fluence in joules is obtained from experimental data in M9196 as the exponential of the dimensionless quantity *k_j_F_j_*, with *F_j_*(λ) being the fluence in a narrow band of UV centered on λ in J/m^2^ and *k_j_*(λ) the decay constant in m^2^/J. The inactivation can also be expressed as a binomial probability. Since the geometric cross-sectional area of the adjacent pyrimidine pairs on the DNA is of the order of nm^2^ (Aksenova *et al.*, 2012) it is convenient to choose area units of nm^2^ for incident photons. Let *F*_ph_(λ) be the fluence [in J/m^2^] corresponding to 1 photon of wavelength λ per nm^2^, given by *hc*/λ, with *h* being the Planck constant, *c* the speed of light, and λ the wavelength of the photon. For example, *F*_ph_(254 nm) corresponds to 0.78 J/m^2^. If α is defined as the dimensionless quantity $\alpha \equiv {k_j} \left( \lambda \right) {F_{{ \rm{ph}}}} \left( \lambda \right)$ and *q* is defined as the probability of no damage, *P*_ND_, resulting from an incident photon (i.e., the probability of repair-deficient spore survival), then *P*_ND_ is given by

\begin{align*}
{P_{{ \rm{ND}}}} = {e^{ - {k_J} \left( \lambda \right) {F_{{ \rm{ph}}}} \left( \lambda \right) }} = {e^{ - \alpha \left( \lambda \right) }} \equiv q \left( \lambda \right) . \tag{5}
\end{align*}

The quantity *q*(λ) is then defined as the probability of all sites within the reference cross-sectional area escaping damage (not absorbing so as to form a dimer lesion) given an incident photon. The probability of damage from the incident photon is then given by *p*(λ) = 1 – *q*(λ), and the result is a simple binomial probability distribution where the probability of exactly *k* damaged sites given *n*_ph_ incident photons is

\begin{align*}
P \left( {k;{n_{{ \rm{ph}}}} , p} \right) = \left( { \begin{matrix} {{n_{{ \rm{ph}}}}} \\ k \\ \end{matrix} } \right) {p^k}{q^{{n_{{ \rm{ph}}}} - k}} , \tag{6}
\end{align*}

and survival for the sensitive strain case requires no damage, *k* = 0 and *P*(survival) = 1 – *P*(0;*n*_ph_,*p*). The probability of damage, *p*, should be proportional to the fraction of sites on the DNA strand susceptible to absorbing a photon of the given energy and forming a dimer, the probability of such a site absorbing a photon and the quantum efficiency of the susceptible site forming a dimer, given absorption of a photon (Jagger, 1976).

To predict inactivation by the continuous solar (or broadband lamp) UV spectrum based on a set of narrow band inactivation measurements requires (1) an IAS for the strain, derived from the narrow band point estimates, which characterizes the inactivation probability as a continuous function of wavelength over the total wavelength range of interest and (2) a method for calculating the total probability of inactivation from broadband irradiation by accounting for contributions to inactivation from all wavelengths in the broadband exposure. M9196 obtained an IAS for the sensitive, repair-deficient strain of *B. subtilis*, TKJ6321, covering the wavelength interval from ∼200 to 400 nm. These data are summarized in Table 1 and plotted in Fig. 1. Attempts to relate UV inactivation action spectra for spores to the general absorption spectra of DNA and other materials within spores have focused largely on water disinfection, considered less than the full solar UV spectrum or nonsolar broadband irradiation, and demonstrated limited success. (Mamane-Gravetz *et al.*, 2005; Keifer, 2007; Chen *et al.*, 2009).

**Table 1.** Data from Munakata *Et Al*. (1991, 1996) for *Bacillus subtilis-*Sensitive Strain TKJ6321 on Surfaces Exposed in Air (1996) and Vacuum (1991)

*Constant*						
λ						
*nm*	*Energy* *eV/phot*	A *Dimensionless*	α *Dimensionless*	p *Probability*	q* = 1 –* p *Probability*	k_j_ *m^2^/J*
190	6.53	0.64	2.75E-02	2.71E-02	9.73E-01	2.63E-02
220	5.64	0.90	5.26E-01	4.09E-01	5.91E-01	5.83E-01
235	5.28	0.94	5.86E-01	4.43E-01	5.57E-01	6.93E-01
255	4.86	1.12	3.72E-01	3.11E-01	6.89E-01	4.77E-01
270	4.59	1.78	6.74E-01	4.90E-01	5.10E-01	9.16E-01
290	4.28	0.50	6.30E-02	6.11E-02	9.39E-01	9.20E-02
300	4.13	0.51	1.33E-02	1.32E-02	9.87E-01	2.01E-02
254	4.88	0.85	3.82E-01	3.18E-01	6.82E-01	4.89E-01
270	4.59	0.86	6.54E-01	4.80E-01	5.20E-01	8.89E-01
290	4.28	0.74	6.74E-02	6.52E-02	9.35E-01	9.84E-02
300	4.13	1.30	1.09E-02	1.08E-02	9.89E-01	1.64E-02
313	3.96	1.21	7.11E-04	7.11E-04	9.99E-01	1.12E-03
334	3.71	1.92	9.52E-06	9.52E-06	1.00E+00	1.60E-05
365	3.40	1.76	1.07E-06	1.07E-06	1.00E+00	1.97E-06
380	3.26	1.16	3.96E-07	3.96E-07	1.00E+00	7.58E-07
400	3.10	0.90	3.85E-08	3.85E-08	1.00E+00	7.75E-08

The first eight values with wavelengths λ from 190 to 300 nm were reported in Munakata *et al.* (1991), where the decay constants, *k_j_* here converted to m^2^/J, were reported in m^2^/10^20^ photons and exposures were done in vacuum. The remainder were reported by Munakata *et al.* (1996), where decay constants were reported in m^2^/J. *A* is the dimensionless constant term multiplying the exponential function fit in M9196 to the data. α is the dimensionless decay constant defined as $= \alpha = {k_j}{F_{{ \rm{ph}}}} \left( \lambda \right)$, where *F*_ph_(λ) is the fluence corresponding to 1 photon of wavelength λ per nm^2^. *q* is the probability of no damage occurring due to an incident photon and 1 – *q* = *p* is the probability of absorption of an incident photon resulting in damage.

A plausible mechanistic functional form for the sensitive repair-deficient *B. subtilis* data can be obtained with the following assumptions: first, the IAS results from photon interaction with several potential types of damage sites, and for each such site type there is a photon energy with the greatest probability of excitation of that site such that it relaxes to a specific damage photoproduct. Second, photons with energies differing from the energy of peak probability of excitation have reduced probability of excitation proportional to ${e^{ - \beta {{ \left\vert {{E_{{ \rm{photon}}}} - {E_{{ \rm{damage}}}}} \right\vert }^2}}}$, where β is some constant, analogous to the Landau-Zener result (Langhals, 2000; Emanuele *et al.*, 2005). Third, the shape of the absorption spectrum for each potential damage site is adequately described by a Gaussian function in energy (Maric and Burrows, 1996).

**FIG. 2.**
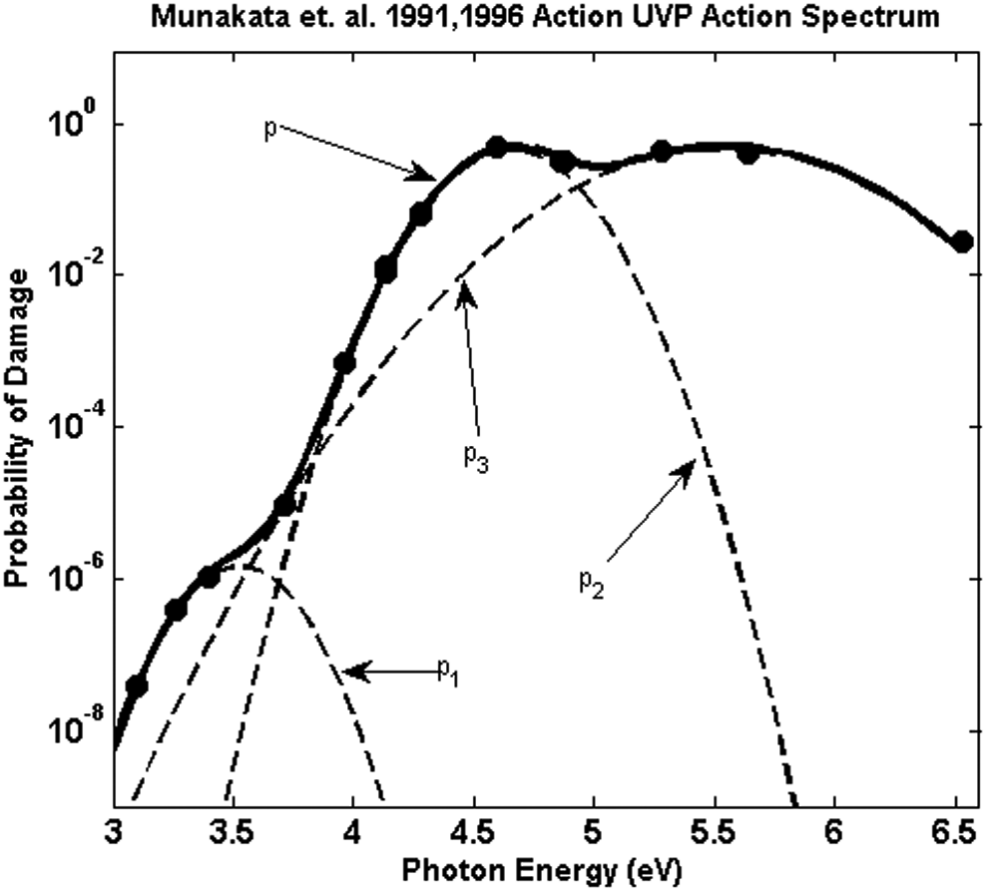
Plot of the values for probability of damage *p*(λ) obtained from M9196 and the Gaussian fit as described in the text. The *dotted curves* labeled *p*_1_, *p*_2_, and *p*_3_ correspond to the Gaussians using the parameters in Table 2. The *solid line*, labeled *p*, is the sum of the probabilities. Damage probability is the probability of damage per photon per nm^2^.

Under these assumptions, for sensitive repair-deficient *B. subtilis* for which a single damaged site results in inactivation, the total damage probability can be expressed as a sum over the probabilities for each potential damage site ${p_{{ \rm{total}}}} \left( \lambda \right) = \mathop \sum \limits_{{ \rm{damage \;types}} \;i} {a_i}{p_i} \left( \lambda \right)$, where *a_i_* represents the relative contribution of each damage mechanism and each of the *p_i_*(λ) are Gaussian probability distributions given by ${ p_i } \left( \lambda \right) = \; \frac { 1 }  { { \sqrt { 2 \pi \sigma _i^2 } } } { e^ { - { \frac { { { \left( { E \left( \lambda \right) - { E_i } } \right) } ^2 } }  { 2 \sigma _i^2 } } } }$, where *E_i_* and σ_*i*_ are, respectively, the energy of peak probability and standard deviations of peak absorption for each damage type *i* and *E*(λ) = *hc*/λ. Nonlinear regression of such Gaussian distributions to the data from M91 (given in Table 1 under the column labeled *p* and plotted in Fig. 2) using the MATLAB (Mathworks, Natick, MA) curve fitting toolbox finds three Gaussians adequately predict the inactivation over the solar wavelength range, with peaks at 3.5, 4.65, and 5.5 eV (351, 267, and 224 nm, respectively). The distribution parameters obtained for the *B. subtilis* exposure data are given in Table 2. Figure 2 plots the M9196 data, the fitted function, and the individual Gaussian components.

**Table 2.** Gaussian Fit Parameters Derived in This Work for Sensitive Repair-Deficient *Bacillus subtilis* Strain Inactivation Action Spectrum Reported by Munakata *Et Al*. (1991, 1996)

*Peak*	*Parameter*		
	a_i_	E_i_	*σ*_i_
	*No units*	*eV*	*eV*
1	1.46E-06	3.53	0.157
2	0.452	4.65	0.187
3	0.495	5.54	0.391

The fractional rms deviation of the spline fit to the Munakata *et al.* (1996) data over the wavelengths from 255 to 300 nm as well as that from the Gaussian fit to the spline fit is <1%. The parameters are those characterizing the probability of damage from an incident photon as a sum over three Gaussian contributions, ${p_{{ \rm{total}}}} \left( \lambda \right) = \mathop \sum \limits_{{ \rm{damage \;types \;}}i} {a_i}{p_i} \left( \lambda \right)$, where ${ p_i } \left( \lambda \right) = \; \frac { 1 }  { { \sqrt { 2 \pi \sigma _i^2 } } } { e^ { - { \frac { { { \left( { E \left( \lambda \right) - { E_i } } \right) } ^2 } }  { 2 \sigma _i^2 } } } }$, and the *a_i_* are constants determined from the Gaussian fit.

Inactivation from a given solar UV exposure expresses the experimentally determined survival of spores exposed for various times in terms of a spore inactivation dose (SID) defined as $${ \rm{SID}} = - \log  \Big (  {N_{e}}   \mathord \big / {N_{c}}
\Big )$$, where *N_e_* is the number of surviving exposed spores and *N_c_* is the number of viable unexposed spores (M96; Munakata *et al.*, 2000). M96 observed that for solar exposure times (about one half hour) in which the irradiance was nearly constant, the survival curves were approximately exponential, so that the fraction of surviving spores is given by $$S \left( t \right) =  {  { {{N_{e}}\left(t\right)}}   \mathord{
\left/ { \vphantom {{{N_e} \left( t \right) } {{N_c}}}} \right.
 { {{N_c}}}} = {e^{ - {
\rm{SIDR}} \times t}}}$$ for time *t* in minutes, where SIDR is the SID rate with $${ \rm{SID}} = { \rm{SIDR}} \times t$$. For repair-deficient spores, prediction of the SID from the IAS is straightforward since the spore must survive all incident photons and the survival probability is given by the IAS directly. In this case, the SID for the sensitive repair-deficient *B. subtilis* is given by either of two equivalent expressions:

\begin{align*}
S \left( t \right) = \;{e^{ - \smallint {k_s} \left( \lambda \right) I \left( \lambda \right) td \lambda }} \equiv {e^{ - { \rm{SIDR}} \times t}} , \tag{7}
\end{align*}

where *k_s_*(λ) is the wavelength-dependent sensitive strain decay constant from the IAS, *I*(λ) is the radiant intensity as a function of wavelength (assumed constant in time over the exposure, so that $I \times t = F , \;{ \rm{the \;fluence}}$), and *t* is the exposure time in minutes for *I* in W/(m^2^-min), or by

\begin{align*}
S \left( t \right) = \; \mathop \prod \limits_ \lambda q{ \left( \lambda \right) ^{60t \times {n_{{ \rm{ph}}}} \left( \lambda \right) }} = \;{e^{ \mathop \sum \limits_ \lambda 60t \times {n_{{ \rm{ph}}}} \left( \lambda \right) { \rm{ln}}q \left( \lambda \right) }} \equiv {e^{ - { \rm{SIDR}} \times t}} , \tag{8}
\end{align*}

where *n*_ph_(λ) is the number of incident photons per second per unit wavelength per unit area, *t* is the time in minutes, the damage probabilities *q*(λ) = 1 − *p*(λ) are obtained as described in Equation (5) and given in Table 3.

**Table 3.** Calculated Spore Inactivation Dose Rate from This Work Compared with Values from Munakata *Et Al.* (1996)

*Insolation*	*SIDR (min^−1^)*			
	*Measured*	*Reference calculated*	*From* q	*From kIt*
Ref. noon	0.385	0.343	0.376	0.376
ASTMG177			0.422	0.422
ASTMG173			0.128	0.128

“From *q*” indicates the SIDR was calculated using probability of photon damage *q* as in the text through $St = \; \mathop \prod \limits_ \lambda q{ \lambda ^{60t \cdot {n_{{ \rm{ph}}}}^ \lambda }} \equiv {e^{ - { \rm{SIDR}} \times t}}$, where *n*_ph_ is the number of photons incident per second at wavelength λ and *t* is the exposure time in seconds and *S*(*t*) is the survival fraction after exposure for *t* seconds. “From kIt” indicates the SIDR was calculated by integrating over fluence as in the text through $S \left( t \right) = \;{e^{ - \smallint {k_s} \left( \lambda \right) I \left( \lambda \right) td \lambda }} \equiv {e^{ - { \rm{SIDR}} \times t}}$, where *k_s_* is the sensitive strain decay constant for fluence in m^2^/J at wavelength λ, *I* is the radiant flux at wavelength λ in W/m^2^, and *t* is the time in seconds.

SIDR, spore inactivation dose rate.

### Modeling repair-capable *B. subtilis* inactivation

**FIG. 3.**
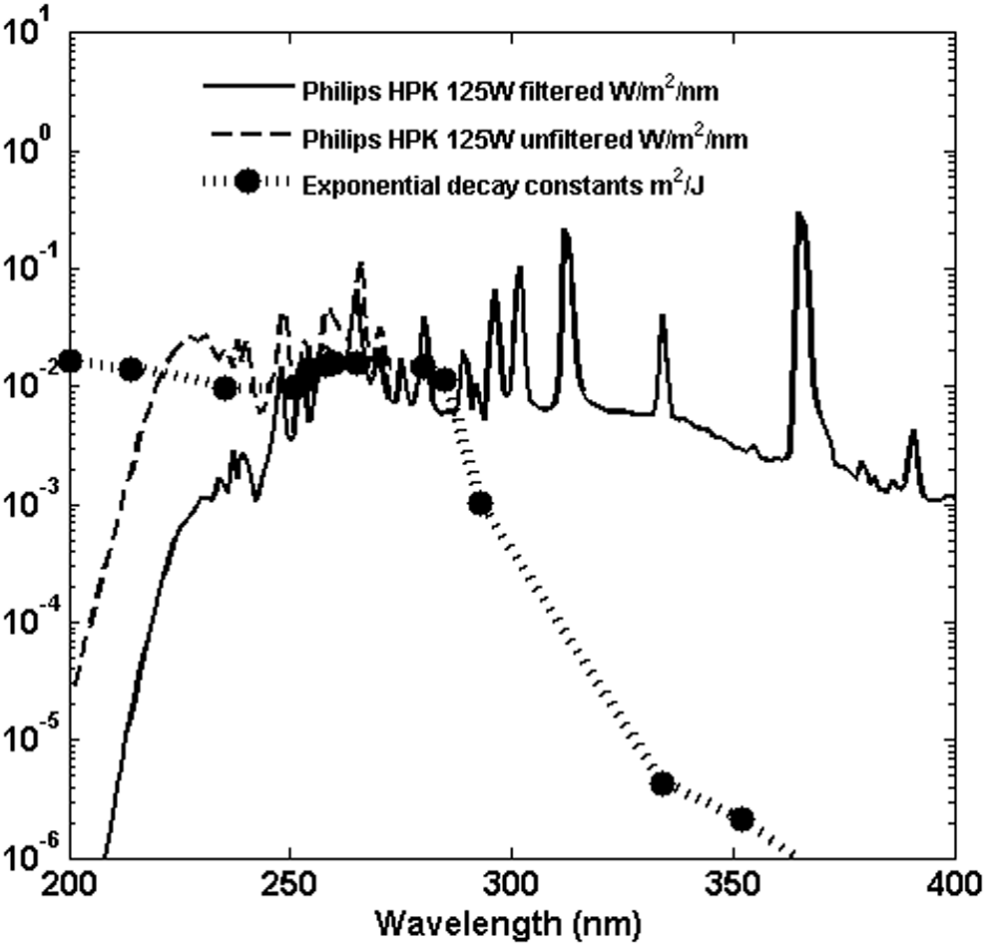
Irradiance spectrum reported in Figure 2 of C2002 for filtered and unfiltered disinfection lamp. Superimposed on that plot are exponential decay constants reported in C2002 for *B. subtilis* ATCC6633.

Solar exposure data for wild-type *B. subtilis* in air have not been reported; however, C2002 reported both a set of narrow band UV exposures and a set of broadband exposures of wild-type *B. subtilis* suspended in water. Figure 3 shows the broadband lamp (Philips HPK 125W) spectral irradiance at the surface of the suspension with and without the Schott WG280 filter used, digitally extracted from Fig. 2 of C2002, and the reported exponential decay constants for *B. subtilis* spores (ATCC6633 wild-type repair-capable strain).

The multitarget expression for decay in the repair-capable case does not provide an immediate, correct probability expression for SID, as was the case for the repair-deficient case above, since if fluence arrives at two narrow wavelength bands, say λ_1_ and λ_2_, then the combined survival rate would be

\begin{align*}
S \left( {{F_1} + {F_2}} \right) & = \Big ( 1 -  { \left( {1 -
{e^{ - {k_1}{F_1}}}} \right) ^{{n_1}}} \Big )  \Big ( 1 - { \left(
{1 - {e^{ - {k_2}{F_2}}}} \right) ^{{n_2}}} \Big ) \\ & \, \ne
\Big ( 1 - { \left( {1 - {e^{ - {k_1}{F_1} + {k_2}{F_1}}}} \right)
^{ \overline n }} \Big),
 \tag{9}
\end{align*}

where the shoulder parameters for wavelengths λ_1_ and λ_2_ are not equal, ${n_1} \ne {n_2}$ (even though the repair capability of *B. subtilis* should be the same for SP regardless of the damaging wavelength, the multitarget function yields a varying value of shoulder parameter *n*) and $\overline n$ is the average value of the shoulder parameter *n*, and the inequality indicates that the two expressions are not equivalent. Accumulating the fluence through $\smallint {k_ \lambda }{F_ \lambda }d \lambda$ inside an expression of the form of the right side of Equation (9) with some averaged value of shoulder parameter *n* is not guaranteed to be equivalent to the accumulated probability over wavelength, even if *n* were constant over the wavelength range, which it is not.

In a binomial representation, the cumulative probability of survival from the combination of flux from each narrow band wavelength interval would be the probability that the sum of damage from both bands would be less than or equal to the amount that can be repaired (Butler and Stevens, 2017):

\begin{align*}
\mathop \sum \limits_{i = 0}^n P \left( {{x_1} + {x_2} = i} \right)
\end{align*}

\begin{align*}
= \mathop \sum \limits_{i = 0}^n \mathop \sum \limits_{ \begin{matrix} {{ \rm{All \;combinations \;}}} \\ {{x_1} , {x_2} \;{ \rm{such \;that \;}}{x_1} + {x_2} = i}  \\ \end{matrix} } \mathop \prod \limits_{ \begin{matrix} {{ \rm{Each \;combination}} \;} \\ {{x_1} , {x_2}{ \rm{ \;such \;that \;}}{x_1} + {x_2} = i} \\ \end{matrix} } B \left( {{x_1} , {N_1} , {p_1}} \right) B \left( {{x_2} , {N_2} , {p_2}} \right) \; , \tag{10}
\end{align*}

where $B \left( {x , N , p} \right)$ is the binomial probability mass function for exactly *x* damaged sites, given *N* incident photons per unit area at the given wavelength intervals with probability *p* of each such photon causing damage, and *x*_1_ and *x*_2_ both range from 0 to *n*, the number that can be repaired. *N*_1_ and *N*_2_ are the number of incident photons in narrow band wavelength range 1 and 2, respectively, and *p*_1_ and *p*_2_ are the damage probabilities for incident photons in each narrow band wavelength range. Equation (10) generalizes naturally to the total wavelength interval concerned as

\begin{align*}
P \left( { \mathop \sum \limits_i {x_i} \le n;{N_i} , {p_i}} \right) = \mathop \sum \limits_{i = 0}^n \mathop \sum \limits_{ \begin{matrix} {{ \rm{All \;combinations \;}}} \\ { \left\{  j \right\}  \;{ \rm{such \;that \;}} \mathop \sum \limits_j {x_{i , j}} = {i_{}}} \\ \end{matrix} } \mathop \prod \limits_{ \begin{matrix} {{ \rm{Each \;combination \;}}} \\ {j \;{ \rm{such \;that}} \; \mathop \sum \limits_j {x_{i , j}} = {i_{}}} \\ \end{matrix} } B \left( {{x_{i , j}} , {N_i} , {p_i}} \right) \; , \tag{11}
\end{align*}

where the index *i* ranges over all wavelength intervals of the incident UV.

## Results

### Dose response of *B. subtilis* spores: repair-deficient strain

An example comparison of the calculated SIDR with experimental results is shown in Table 3 for the data reported in Munakata *et al.* (1996) from exposures to solar UV of repair-deficient *B. subtilis* made on July 28, 1993, and given in Fig. 3 and Table 2 of that reference. Table 3 here shows the measured SIDR and values calculated using the action spectrum in Table 2 of this work and the measured solar spectral irradiance given in Fig. 3 of M96 for the noon exposure for that date.

### Dose response of *B. subtilis* spores: repair-capable strain

Survival probability of wild-type *B. subtilis* spores capable of repairing up to *n* damaged sites per nm^2^ given by Equation (11) can be calculated efficiently using MATLAB intrinsically vectorized features. Let the total incident flux, from wavelengths λ_min_ to λ_max_, be divided into *L* wavelength bands each centered on wavelengths λ_*i*_, with $i \in I = \left\{  {1 , 2 , 3 \cdots L} \right\}$, in each of which the number of incident photons per unit time is *m_i_* and the IAS provides *p_i_* the probability of damage from an incident photon in that wavelength band with probability of no damage *q_i_* = 1 − *p_i_*. A single MATLAB command generates the array of so-called *n*-combinations with repetitions:

\begin{align*}
{ \mathbb C_{L , n \;}} \left( {k , j} \right) = \left[ { \begin{matrix} {{c_{11}}{c_{12}} \cdots {c_{1n}}} \\ \vdots \\ {{c_{ \cal M1}}{c_{ \cal M2}} \cdots {c_{ \cal M n}}} \\ \end{matrix} } \right] , \tag{12}
\end{align*}

where the *c_k,j_* are elements of *I*, possibly repeated, and $$\cal M = \left( { \begin{matrix} {L + n - 1} \\ n \\ \end{matrix} } \right)$$, the number of combinations of *L* objects taken *n* at a time with repetitions.

Each row of $\mathbb C \left( {k , j} \right)$ is then one possible combination of the $\cal M$ possible combinations, and represents one possible damage outcome for the incident photons. For illustration, if *L* = 3 and *n* = 2,

\begin{align*}
{ \mathbb{C}_{3 , 2}} \left( {k , j} \right) = \left[ { \begin{matrix} { \begin{matrix} 1 & 1 \\ 1 & 2 \\ 1 & 3 \\ \end{matrix} } \\ { \begin{matrix} 2 & 2 \\ 2 & 3 \\ 3 & 3 \\ \end{matrix} } \\ \end{matrix} } \right] \tag{13}
\end{align*}

From $\mathbb{C} \left( {k , j} \right)$ an *L* by ${\cal M}$ array, ${ \mathbb{C}_{L , n \;{ \rm{row}}}}$, can be constructed, again with a single MATLAB command, with *L* entries in each row corresponding to each of the *L* wavelength intervals and each row corresponding to one of the $\cal M$ possible combinations. Each row entry is either 0 or the number of occurrences of the *i*th wavelength interval index in the corresponding row of $\mathbb C \left( {k , j} \right)$. For the illustrative *L* = 3, *n* = 2 case

\begin{align*}
{ \mathbb C_{3 , 2 \;{ \rm{row}}}} \left( {i , j} \right) = \left[ { \begin{matrix} { \begin{matrix} 2 & 0 & 0 \\ 1 & 1 & 0 \\ 1 & 0 & 1 \\ \end{matrix} } \\ { \begin{matrix} 0 & 2 & 0 \\ 0 & 1 & 1 \\ 0 & 0 & 2 \\ \end{matrix} } \\ \end{matrix} } \right] \tag{14}
\end{align*}

Then the probability of exactly *n* damage events corresponding to each occurrence of each combination represented by the *j*th row in ${\mathbb C}_{L , n \;{ \rm{row}}} \left( {i , j} \right)$ can again be evaluated with a single MATLAB command, and is

\begin{align*}
\begin{split}{P_j} & =  P \left( {n \;{ \rm{damage \;events \;due}}
\;{j^{{ \rm{th}}}}{ \rm{row}}} \right) \\& = \mathop \prod
\limits_{i = 1}^L B \left( {{ \mathbb C_{L , n \;{ \rm{row}}}}
\left( {i , j} \right) , {m_i} , {p_i}} \right) \; \; \;
,\end{split}
 \tag{15}
\end{align*}

where *B* is again the binomial probability mass function as used in Equation (10). In illustrative 3, 2 case this would yield, for *j* = 1, the first row:

\begin{align*}
\begin{split}{P_1} = P \left( {2 \;{ \rm{damage \;events \;due}}
\;1{\rm{st}} \;{ \rm{row}}} \right) \\= B \left( {2 , {m_1} ,
{p_1}} \right) B \left( {0 , {m_2} , {p_2}} \right) B \left( {0 ,
{m_3} , {p_3}} \right) .\end{split}
 \tag{16}
\end{align*}

Explicitly written out for illustration, *P*_1_, is

\begin{align*}
{P_1} = \left( { \begin{matrix} {{m_1}} \\ 2 \\ \end{matrix} } \right) p_1^{2}{q^{{m_1} - 2}} \times \left( { \begin{matrix} {{m_2}} \\ 0 \\ \end{matrix} } \right) p_1^0{q^{{m_2}}} \times \left( { \begin{matrix} {{m_3}} \\ 0 \\ \end{matrix} } \right) p_1^0{q^{{m_3}}} \tag{17}
\end{align*}

The total probability of the occurrence of exactly *n* damage events given *m_i_* photons incident in the λ_i_ wavelength intervals, each with probability of damage per photon *p_i_*, is then the sum over all *P_j_*, which is again done with a single MATLAB command:

\begin{align*}
P \left( {n;{m_i} , {p_i}} \right) = \mathop \sum\limits_j {P_j} (
n;{m_i}{p_i} ) = \mathop \sum \limits_{j = 1}^{\cal M} \mathop
\prod \limits_{{i = 1}^L B} \left(  \mathbb {C_{L , n \;{
\rm{row}}}} \left( {i , j} \right) , {m_i} , {p_i} \right).
\tag{18}
\end{align*}

The probability of survival given that up to *n* damaged sites can be repaired is then

\begin{align*}
\mathop \sum \limits_{s = 0}^n P \left( {s;{m_i} , {p_i}} \right)
= \mathop \sum \limits_{s = 0}^n \mathop \sum \limits_j {P_j} (
s;{m_i}{p_i} ) \\= \mathop \sum \limits_{s = 0}^n \mathop \sum
\limits_{j = 1}^{\cal M} \mathop \prod \limits_{i = 1}^L B \left(
{{ \mathbb C_{L , s \;{ \rm{row}}}} \left( {i , j} \right) , {m_i}
, {p_i}} \right) .
 \tag{19}
\end{align*}

Equation (19) is the computational form of Equation (11).

Damage probabilities as a function of wavelength were estimated from the exponential decay rates given in C2002 (plotted in Fig. 3), by setting $q = {e^{  -  \alpha }}$ and letting *p* = 1 – *q*, as discussed above. The values obtained are given in Table 4. C2002 measured inactivation and reported raw data for exposures to 6 quasi-monochromatic sources at 214, 254, 251, 293, 334, and 352 nm, with source bandwidths of 20 nm (see C2002 for a description of the sources and methods). The remaining values in Table 4 were obtained by and reported in C2002 from the literature. A cubic spline over wavelength was used to interpolate between values.

**Table 4.** Data from Cabaj *Et Al*. (2002) for *Bacillus subtilis* Wild-Type Repair-Capable Strain (ATC6633) Exposed in Water Suspension to Narrow Band Irradiation at Wavelengths λ

*Decay constants and probabilities*				
*λ (nm)*	k_j*,10*_	k_j,e_	p_*ph*_	p_ph_^*^
200	7.16E-03	1.65E-02	1.62E-02	3.25E-02
214	6.23E-03	1.43E-02	1.32E-02	2.65E-02
235	4.28E-03	9.86E-03	8.30E-03	1.66E-02
251	4.28E-03	9.86E-03	7.78E-03	1.56E-02
254	6.12E-03	1.41E-02	1.10E-02	2.19E-02
258	6.73E-03	1.55E-02	1.19E-02	2.37E-02
260	6.98E-03	1.61E-02	1.22E-02	2.44E-02
265	7.04E-03	1.62E-02	1.21E-02	2.41E-02
280	6.43E-03	1.48E-02	1.04E-02	2.09E-02
285	4.90E-03	1.13E-02	7.83E-03	1.57E-02
293	4.47E-04	1.03E-03	6.97E-04	1.39E-03
334	1.88E-06	4.33E-06	2.57E-06	5.15E-06
352	9.18E-07	2.11E-06	1.19E-06	2.39E-06
400	5.20E-08	1.20E-07	5.95E-08	1.19E-07

Cabaj *et al.* (2002) reported results in base 10, that is, survival ∼10^−*kF*^, where *F* is fluence reported in joules and *k* is the decay constant. These values are indicated in the table as *k_j_*_,10_ and have units m^2^/J. The values denoted *k_j,e_* are simply converted to the more widespread base *e*, that is, where Survival ∼*e*^-*kF*^ and also have units m^2^/J. The values denoted *p*_ph_ are the probabilities that a single photon incident per nm^2^ will result in damage obtained as described in the text. The values denoted *p*_ph_^*^are simply two times *p*_ph_ as discussed in the text.

**FIG. 4.**
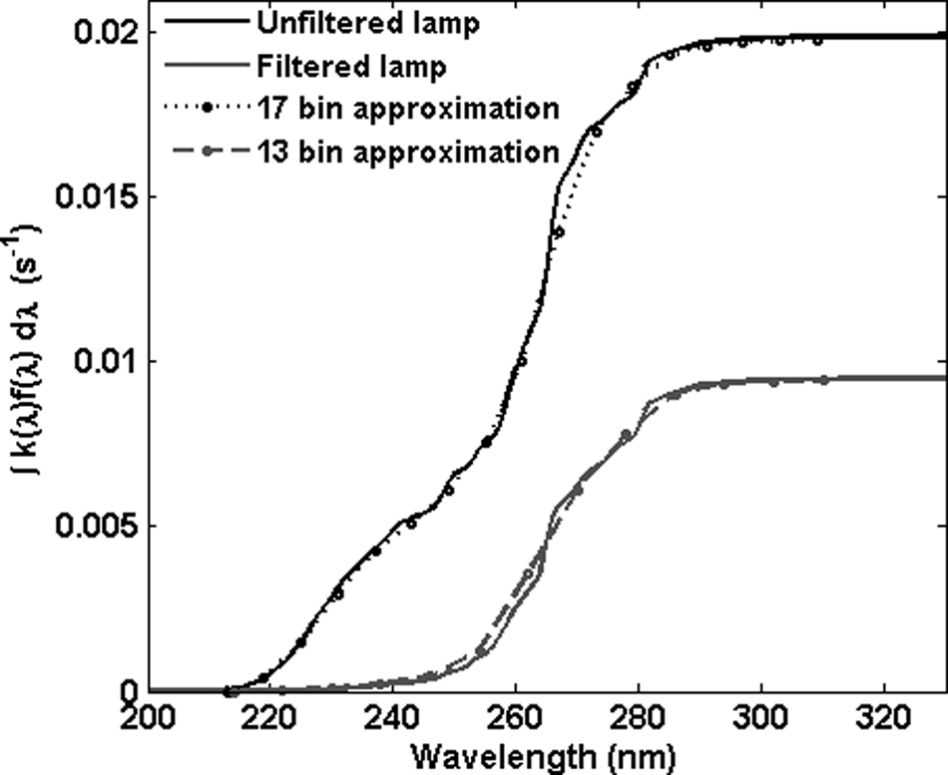
Cumulative inactivation action $\int {{f_ \lambda }{k_ \lambda }{d_ \lambda }}$ or the *B. subtilis* spores exposed in water to the filtered and unfiltered lamp as reported in C2002. *Solid lines* are calculated from the data reported in Figure 7, *dots* are representative points calculated as described in the text.

If the wavelength intervals are chosen to be 1 nm wide over the full range from 200 to 400 nm, the computation of Equation (19) is impractical, since the number of *k*-combinations of 200 intervals taken up to, for example, 4 at a time would be $$\left( { \begin{matrix} {200} \\ 4 \\ \end{matrix} } \right) \sim \;6.5e7$$. The computation is made practical by considering the net inactivation over wavelength, plotted in Fig. 4, for the unfiltered and filtered lamp spectra. The solid lines in the plot show the cumulative integral of the product $k \left( \lambda \right) \times f \left( \lambda \right)$, which gives the net inactivation per second; that is, the term that would appear in an exponential expression for survival. More than 99% of the inactivation occurs over a relatively small wavelength interval, from ∼230 to 310 nm for the filtered lamp and from ∼200 to 310 nm for the unfiltered lamp. The dots plotted in Fig. 4 show the effect of binning the fluence into 6 nm intervals, calculating the inactivation weighted decay constant for each interval and multiplying the fluence over the interval. For the filtered exposures, 13 wavelength bins match the cumulative inactivation closely. For the unfiltered exposures, 17 bins were used.

Determining the appropriate binomial damage probabilities for the repair-capable case is less straightforward than for the repair-deficient case. In the repair-deficient case, the simple exponential decay in fluence is mathematically equivalent to the binomial probability if the units are simply changed from joules per unit area to photons per unit area. In the repair-capable case, the multitarget expression, especially with noninteger shoulder parameter, is not mathematically equivalent to expressions with fixed integer *n*, such as a series event or a cumulative binomial distribution, and derived decay constants can differ by as much as a factor of 2 or 3 [see the discussion above comparing the results of Ke *et al.* (2009) and Lim and Blatchley (2009) for *B. subtilis* strain ATC6633]. As a result, the estimates obtained by simple unit change of the multitarget decay constants may be less than those obtained by simple conversion of the data given in C2002, as shown here in Table 4.

Therefore, a simple iterative procedure was done in which the damage probabilities calculated directly from the decay constants in C2002, *p*_ph_ in Table 4, were multiplied by factors ranging from 1.5 to 2.5 in increments of 0.05, and Equation (19) was used to generate predicted survival according to the irradiance of the filtered and unfiltered lamp and given in C2002, with wavelength binning as described above. For each iteration, the sum of the squared differences between the prediction and the data was calculated for *n* from 3 to 7, with the result that a factor of 2.0 minimized the residual sums for both the filtered and unfiltered exposures with *n* = 6. The values listed in Table 4 labeled *p*_ph_* are the resulting binomial probabilities used. Calculations were done on a Dell Optiplex Tower 5000 series with 64 GB memory and required ∼20 s per exposure.

**FIG. 5.**
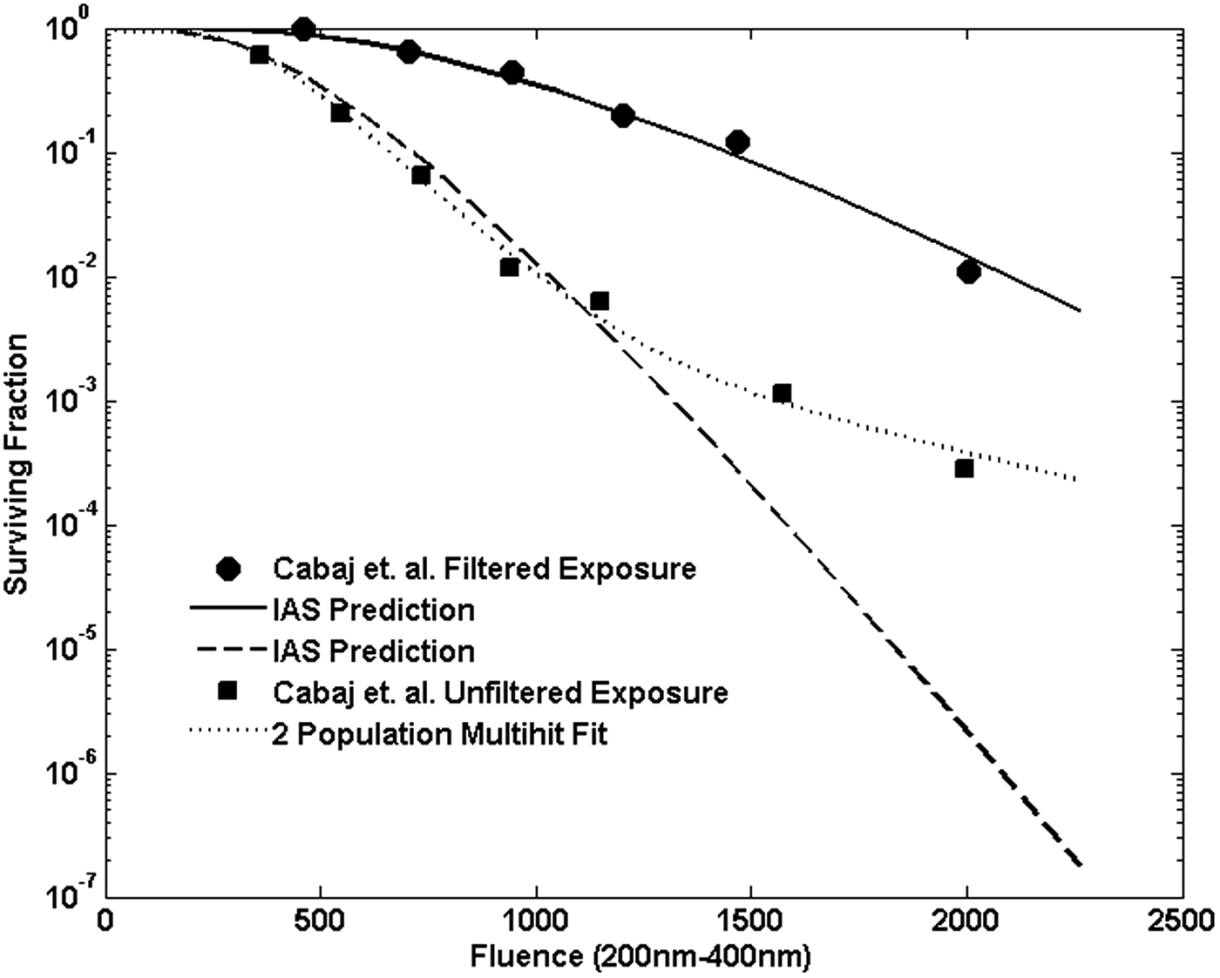
Survival predicted for broadband exposures to the unfiltered and filtered lamp compared with the measured values. Fluence values are total fluence integrated from 200 to 400 nm.

**FIG. 6.**
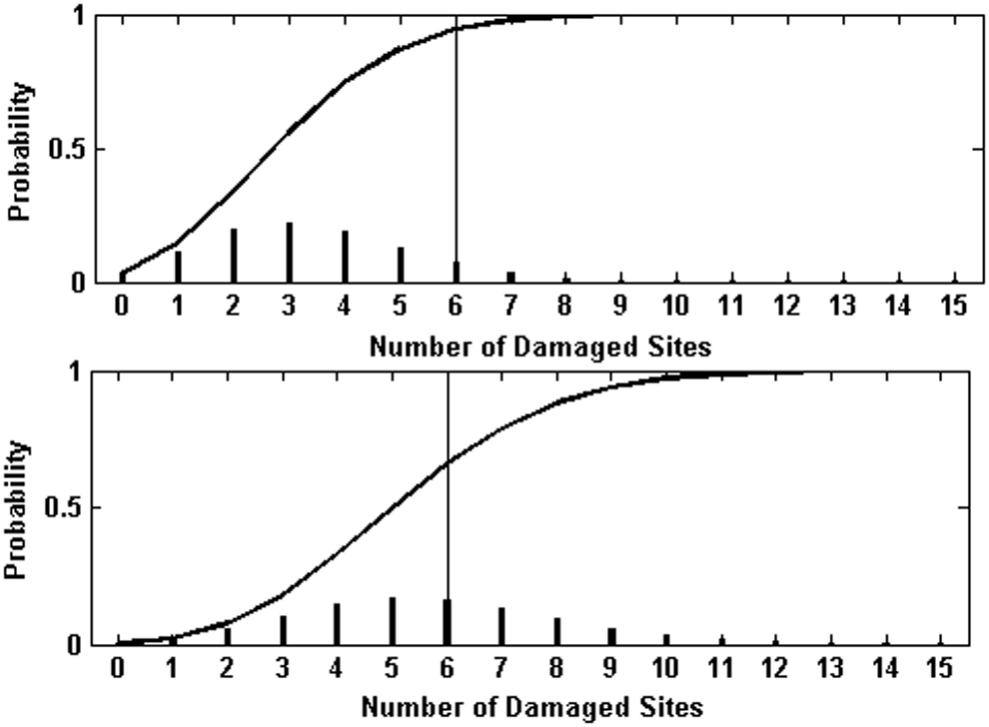
Binomial probabilities of damage at levels on *n* from 0 to 15 for 3 and 5 min exposures to the unfiltered lamp. The intersection of the *vertical line* with the cumulative probability plotted as a *soling line* indicates the probability of survival if damage can be repaired up to the six site level.

Resulting predicted inactivation for the filtered and unfiltered exposures is shown in Fig. 5. In both cases the agreement with the shoulder behavior and initial decay rate is good. However, at larger fluences, the experimental inactivation rate appears less than the predicted, particularly in the unfiltered case. For comparison, the dotted line in Fig. 5 shows a standard two-population multitarget fit to the unfiltered data. Figure 6 shows the damage probability distributions for 3 and 5 min exposures to the filtered lamp, which illustrates the evolution of the net inactivation with exposure time at a damage level *n* = 6.

The above treatments assume that the probability of damage for an incident photon does not change as damage is accumulated. However, neither a binomial nor multitarget approach naturally accounts for the observed fact for narrow band exposures that the decay rate, in the fluence region in which decay is exponential, of repair-capable strains is significantly lower than the exponential decay rate for repair-deficient strains prepared and exposed under the same conditions. This means that fitting such functions (binomial or multitarget) to the sensitive strain inactivation and then using the sensitive strain decay constants to predict the repair-capable strain, allowing only the capability of repair, that is, the shoulder parameter *n*, to vary, do not adequately represent the behavior of the repair-capable strain.

**FIG. 7.**
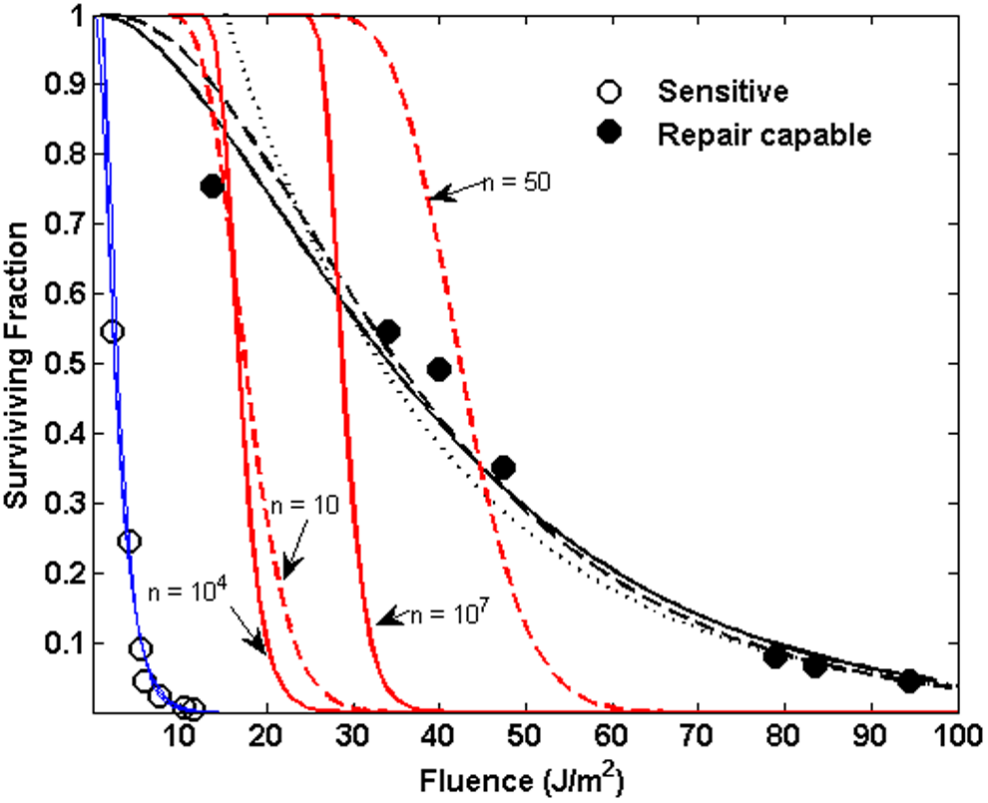
Comparison of exponential, multitarget, and binomial functions for predicting inactivation from 255 nm irradiation of sensitive strain and repair-capable strain *B. subtilis*. *Open blue circles* plot the inactivation reported in M91 for sensitive, repair-deficient strain *B. subtilis. Filled black circles* plot the inactivation reported in M91 for the wild-type repair-capable strain. *Blue lines* plot fitted exponential (*dotted line*), multitarget (*solid line*), and binomial (*dashed line*) function fits to the sensitive strain data. *Black lines* plot similar function fits to the repair-capable strain, allowing the inactivation constant to vary. *Solid red lines* plot multitarget expressions using the sensitive strain decay constant, varying only the shoulder parameter *n*. Values with text *arrows* indicate the multitarget and binomial shoulder parameters used in the respective functions.

Figure 7 illustrates the situation for the 255 nm inactivation data from M91. The sensitive strain is plotted as blue circles, and the blue lines show the results of fitting a simple exponential, a multitarget, and a binomial model to the data, all of which provide fitted curves with accuracies commensurate to the variations in the experimental data. The red solid and dashed lines show the result of keeping the decay constants fixed at the values obtained by fitting to the sensitive strain data and allowing only the shoulder parameters to be increased. Solid red lines correspond to the multitarget function, and dashed lines correspond to the binomial function, from which it is clear that allowing for repair alone cannot adequately match the repair-capable strain inactivation. The black solid line (multitarget fit), dashed line (binomial fit), and dotted line (exponential fit) show that the functions can adequately fit the repair-capable behavior, but only if the inactivation constant is allowed to vary.

The narrow band inactivation constant should vary as damage is accumulated since the number of available sites for damage is fixed by the number of occurrences of susceptible sites on the base pair sequence. In this case the probability of damage by the *n*th photon should be dependent on how many damage sites have been incurred by previous photons, since those lesions deplete the number of available lesion-forming sites. The dependence of damage probability on increasing levels of damage was measured for *B. subtilis* obtained by direct exposure of dry spores (Douki *et al.*, 2005). In particular, the number of lesions (intrastrand SP, interstrand SP, and TT-CPD) resulting from varying levels of UVC fluence was measured for dry spores and plotted in Fig. 2 of Douki *et al.* (2005).

These data are well fit by simple power laws with ${ \rm{Number}} \,{ \rm{of}} \,{ \rm{lesions}} \, = \,a{n^b}$, where *a* and *b* are constants and *n* is the number of UVC photons/nm^2^. For the data of Douki *et al.* (2005), the resulting constants are *a* = 260, 3, 0.35 total lesions for the 4.2e6 base pairs and *b* = 0.67, 0.65, 0.61 for the intrastrand SP, interstrand SP, and TT-CPD formation, respectively. This indicates that the probability of incurring damage with additional incident photons decreases as the amount of damage increases, with damage increasing roughly as number of incident photons to the 2/3 power. Fluences applied in Douki *et al.* (2005) only extended down to ∼290 photons/nm^2^; however, if the power law behavior is extended down to the value at which the shoulder onset occurs in *B. subtilis*, ∼50 photons/nm^2^, it indicates that ∼3,500 intrastrand SP dimers would be formed by that fluence level. This value is in rough accord with the capacity of *B. subtilis* spore photoproduct lyase (SPL) to repair (Yang and Li, 2015), which indicates that there are up to 200 SPL molecules in each spore, each of which repairs ∼0.35 min^−1^ dimers, so that a 60 min germination time has a repair capability of ∼4,200 SP sites.

**FIG. 8.**
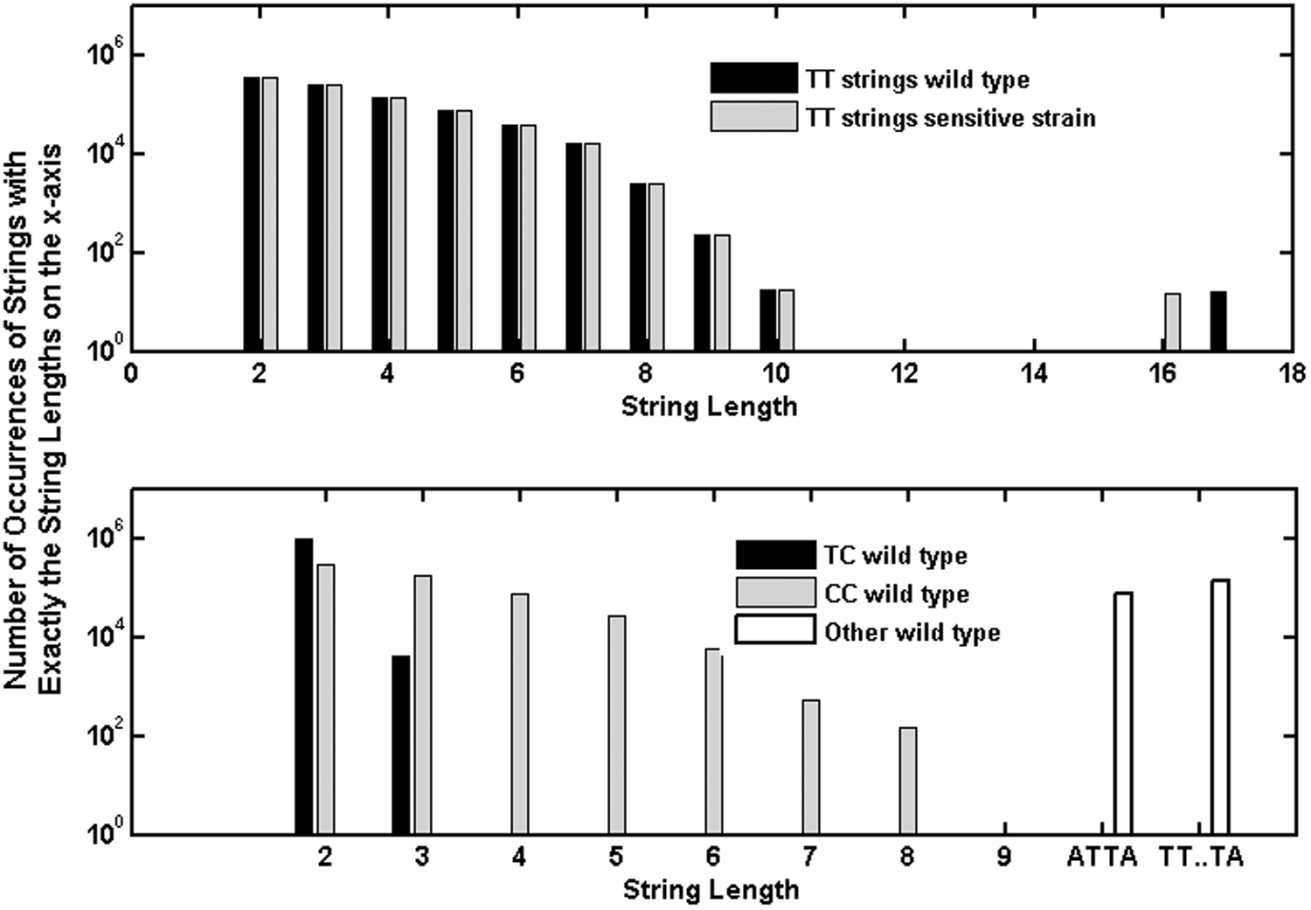
*Upper plot* compares the numbers of occurrences of strings of thymines of various lengths in the gene sequence of wild-type *B. subtilis* with those of the sensitive strain. *Lower plot* shows the numbers of occurrences of various other base pair strings in the wild-type gene sequence.

As shown in Fig. 1, the decay constants for repair-capable *B. subtilis* reported in M9196 for wavelengths between 220 and 270 nm are about a factor of 10 less than those reported for the sensitive strain at the same wavelengths. This is despite the fact that the genes excised to form the sensitive strain amount to only ∼0.2% (7.5e3 bp) of the total number of base pairs (4.2e6) in the *B. subtilis* genome (Kunst *et al.*, 1997), and the distribution of occurrences of TT sequences differs by less than ∼0.1% (Fig. 8). The numbers of other relevant features such as TC, CT, and TT strings flanked by adenines such as (ATTA, ∼75,000 occurrences or any length T string T…TA, ∼1.3e5) differ by about the same percentages between the two strains. Both strains have the same SASP and DPA components, and were exposed under the same conditions, which should result in A conformation DNA (Setlow, 1992).

The only mechanistic difference between the inactivation of the two strains is that the number of lesions necessary for inactivation is larger for the repair-capable strain since it must exceed the number that can be repaired later, during germination, suggesting that the probability of damage from an incident photon decreases as the number of lesions increases.

A mechanistic model based on photon flux instead of energy fluence can be derived, which incorporates the concept of varying damage probability with increasing damage, by assuming that the probability that the *n*th photon incurs damage depends on the order in which the photons are incident; that is, before or after previous damage. The resulting probability of damage can be calculated by enumerating the possible combinations. The probability of the first damage incurred can be denoted *p*_1_, the second *p*_2_, the third *p*_3_, and so on with *p*_1_ > *p*_2_ > *p*_3_. The probability of survival given 0, 1, or 2 previous damage sites is then *q*_1__=_ 1 – *p*_1_, *q*_2__=_ 1 – *p*_2_, and *q*_3__=_ 1 – *p*_3_, with *q*_1_ < *q*_2_ < *q*_3_.

**FIG. 9.**
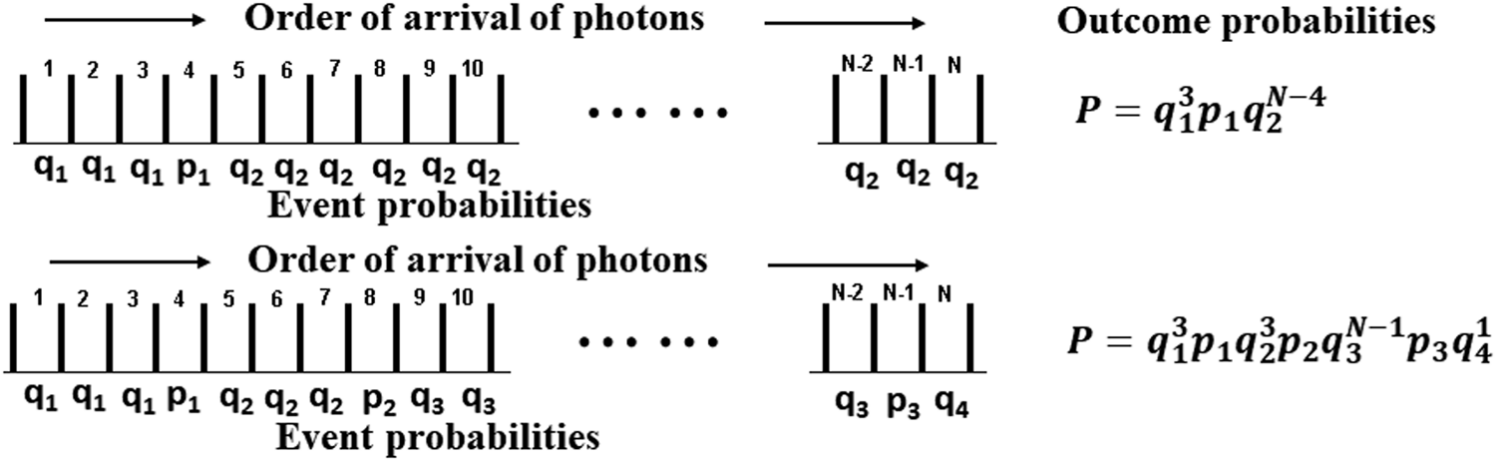
Arrival orders for *N* photons for two cases of possible combinations of damage occurrence. The values of the probabilities and relationship between them are discussed in the text.

Figure 9 illustrates two possible combinations of damage orders. In the upper case *N* photons have been incident, and one damage has occurred after three previous incident photons failed to incur damage. The probability of surviving those three photons is *q*_1_^3^, and the probability of surviving the *N* − 4 photons after damage occurs is *q*_2_^*N*−4^. The probability of this particular outcome is $P = {p_1}q_1^3q_2^{N - 4}$. In the second case *N* photons are incident, and damage occurs on photons 4, 8, and *N* − 1. The probability of this occurrence is $P = {p_1}{p_2}{p_3}q_1^3q_2^3q_3^{N - 1}q_4^1$. Enumerating the cases and summing over all possible combinations yield *P*(*i*;*N*), the probability of *i* damage lesions occurring from *N* incident photons, as

\begin{align*}
P \left( {0;N} \right) = q_1^N , \;
\end{align*}

\begin{align*}
P \left( { 1;N } \right) = { p_1 } \mathop \sum \limits_ { i = 0 } ^ { N - 1 } q_1^iq_2^ { N - 1 - i } = { p_1 } { \frac { \left( { q_2^N - q_1^N } \right) }  { \left( { { q_2 } - { q_1 } } \right) } } 
\end{align*}

\begin{align*}
&  P \left( { 2;N } \right) \; = { { { p_1 } { p_2 } }  {
\left( { { q_3 } - { q_2 } } \right) } } \mathop \sum \limits_ { i
= 0 } ^ { N - 2 } q_1^i \left( { q_3^ { N - 1 - i } - q_2^ { N - 1
- i } } \right) \\ & \, \, \, \, \, \, \, \, \, \, \, \, \, \, \,
\, \, \, = { \frac { { p_1 } { p_2 } }  { \left( { { q_3 } - { q_2
} } \right) } } \left\{ { { \frac { { q_3 } \left( { q_3^ { N - 1
} - q_1^ { N - 1 } } \right) }  { { q_3 } - { q_1 } } } - { \frac
{ { q_2 } \left( { q_2^ { N - 1 } - q_1^ { N - 1 } } \right) }  {
{ q_2 } - { q_1 } } } } \right\}
\end{align*}

\begin{align*}
P \left( { 3;N } \right) = { \frac { { p_1 } { p_2 } { p_3 } }  { \left( { { q_4 } - { q_3 } } \right) } } \mathop \sum \limits_ { k =\; 0 } ^ { N - 3 } q_1^k \mathop \sum \limits_ { m = k + 2 } ^ { N - 1 } q_2^ { m - k - 2 } \left( { q_4^ { N - m } - q_3^ { N - m } } \right) \tag { 20 } 
\end{align*}

The probability of damage being less than or equal to *n* is then $P \left( {i \le n;N} \right) = \mathop \sum \limits_{i = 0}^n P \left( {i;N} \right)$. The sums for cases *i* = 1 and 2 have been done using the partial geometric sum identity $\mathop \sum \limits_ { t = 0 } ^ { n - 1 } { r^t } = { \frac { 1 - { r^n } }  { 1 - r } } \; \;$. In the limit of constant damage probability, that is, ${p_i} \to p + h$ as $h \to 0$, the expressions become the corresponding terms in a simple binomial distribution with constant *p*.

**FIG. 10.**
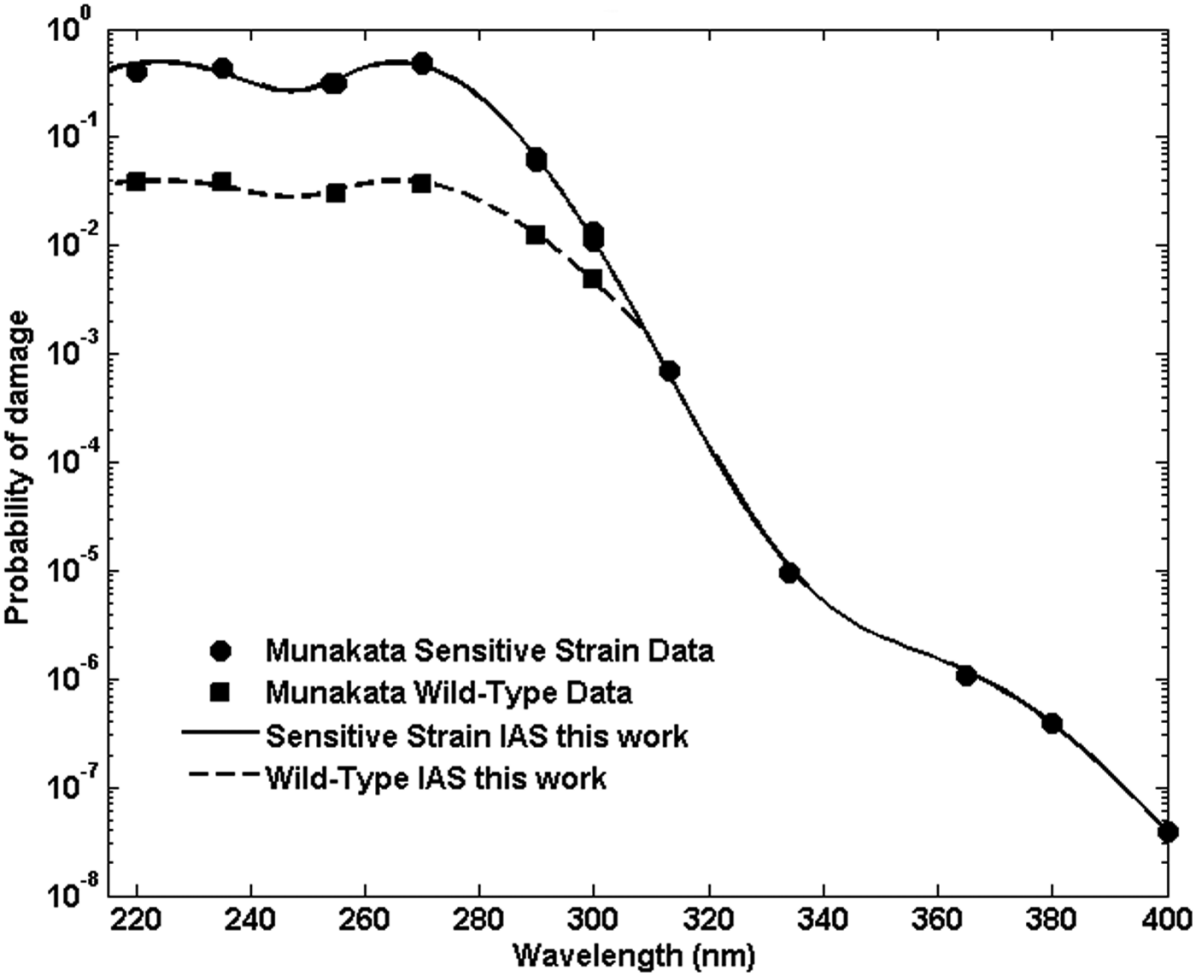
Inactivation action spectrum as derived in the text for sensitive and wild-type *B. subtilis* strains from the data reported in M9196. Probabilities are reported in probability per incident photon per nm^2^.

The survivability of wild-type *B. subtilis* spores reported by M91 at wavelengths relevant to solar exposure (255, 270, 290, and 300 nm) was fit using nonlinear regression to Equation (20), and the results are given in Table 5. The data were digitally converted from the plots in M91, and regressions were done in two ways: first allowing *p*_1_ to be a free parameter determined by the regression, and second setting *p*_1_ equal to the sensitive strain value obtained from the M91 data given in Table 1. The final probability values (*p*_3_ in this case) accord reasonably well with the values given in Table 4 for the wild-type damage probabilities. The wild-type damage probabilities, *p*(wt), are well described by a power law in the sensitive strain probabilities, *p*(ss), as ${p_{{ \rm{wt}}}} = p \left( {{ \rm{wt}}} \right) = { \rm{ap}}{ \left( {{ \rm{ss}}} \right) ^b}p_{{ \rm{ss}}}^b$, where *a* = 0.0595 and *b* = 0.565. This relation has *p*(ss) = *p*(wt) at *p*(wt) = 0.0015. If the IAS given by the parameters in Table 2 is modified to set $p \left( {{ \rm{wt}}} \right) = { \rm{ap}}{ \left( {{ \rm{ss}}} \right) ^b}$ for *p*(ss) <0.0015, the resulting wild-type IAS is plotted in Fig. 10, along with the data from M9196 for sensitive strain and wild-type narrow band exposures.

**Table 5.** Regression Results for Conditional Probability Model for Data Given in Munakata *Et Al*. (1991)

*λ (nm)*	p*_1_ unconstrained*			p*_1_ set to sensitive strain value*		
	p_*1*_	p_*2*_	p_*3*_	p_*1*_	p_*2*_	p_*3*_
255	3.58E-01	3.90E-02	2.85E-02	3.11E-01	4.79E-02	3.36E-02
270	7.69E-01	6.37E-02	4.14E-02	4.90E-01	5.31E-02	4.44E-02
290	7.13E-02	1.71E-02	1.63E-02	6.52E-02	1.70E-02	1.63E-02
300	2.21E-02	5.89E-03	5.84E-03	1.32E-02	4.83E-03	n/a

For each wavelength λ, *p*_1_ is the probability of an incident photon resulting in damage to the DNA, given that no previous damage has occurred. *p*_2_ is the probability of an incident photon resulting in DNA damage, given that one previous photon has resulted in damage. *p*_3_ is the probability of an incident photon resulting in DNA damage, given that two previous photons have incurred damage.

## Discussion

M91 and M96 provided sufficient narrow band data as well as solar exposures for the *B. subtilis* sensitive strain that either a simple cubic spline data look-up or the Gaussian parameters in Table 2 provide equivalent estimates at any wavelength in the relevant solar UV spectrum, with negligible difference in accuracy or computational burden. Either can be used to obtain estimates of SID commensurate with observational uncertainties. Also, either the exponential or the binomial integration procedures provide equivalent values for SID. The primary potential advantage of the Gaussian form is to use for potential curve fitting for other spores similar to *B. subtilis* for which data is available for less extensive wavelengths, such as *B. anthracis* (for biohazard assessment) or *B. thuringiensis* (for biopesticide effectiveness evaluation).

Conditional damage probabilities given in Equation (20) are unwieldy for extension to higher orders of damage levels as the expressions become algebraically complicated and require a relatively large number of parameters for fitting to data. The primary utility of the approach is to provide quantitative insight into the dependence of the apparent decay constant on the level of damage. Since that dependence is apparently based on the number of available damage sites as a function of wavelength as well as the level of damage that can be repaired, it suggests that by including such factors it may be possible to improve accuracy in genomic predictions of UV decay across spore-forming bacterial species and for various wavelengths (Kowalski, 2009, 2011).

The joint binomial approach of Equation (11) provides an estimate consistent with a mechanistic view of the damage and repair processes as well as being consistent with the accumulation of probability of damage over wavelength. It does not require modifying or using an average value for the shoulder parameter representing the damage level as was done in C2002. Instead the damage level, *n* in the binomial, is the same across all wavelengths. The approach also accords well with the experimental broadband exposure data given in C2002. The deviation seen in Fig. 5 for the unfiltered case, in which there appears to be a two-stage decay, may indicate the presence of either a resistant subpopulation or clumping of spores in the suspension, which could result in shielding of some spores from the UV fluence (Cerf, 1977). Such tailing has been observed in other water suspension exposures (Pennell *et al.*, 2008). This suggests that evaluation of water disinfection systems should consider the magnitude of possible effects of tailing, particularly at high dosages or high levels of disinfection.

Since the conditional probability approach indicated that the damage probability may change as the level of damage increases, the adjustments applied to the decay probabilities, in Table 4, obtained from multitarget regression could reflect this effect as well. If so, the damage probabilities used in the binomial approach may represent an average over the probabilities at low and high damage levels.

Joint binomial approach of Equation (11) was applied here only to the water suspension exposures but could easily be extended to exposures of repair-capable *B. subtilis* spores or other species such as *B. anthracis* or *B. thuringiensis* spores in air or on surfaces. At the present time, data for broadband or solar exposures of these species are not available. Demonstration of the applicability of the approach to such exposures could be useful for hazard assessments (*B. anthracis*), or evaluating or optimizing biopesticide performance (*B. thuringiensis*).

## Conclusion

This study has demonstrated a mechanistic approach to predict inactivation of *B. subtilis* spores using a cumulative binomial distribution for damage incurred by incident photons based on the probability of damage per incident photon. The approach allows accumulation of damage over wavelength in a probabilistically consistent manner to estimate survival of spores irradiated by broadband and solar UV. The approach yields more accurate prediction of broadband inactivation of spores, and provides a direct quantitative link of survival to photochemically based probabilities of damage site formation and the relationship of damage probabilities to the amount of damage sustained. The approach provides more accurate estimates of water disinfection effectiveness, and can provide similar assessments of survival of spores exposed in air to broadband or solar UV. In addition, the approach naturally predicts the reduced inactivation rate constants in repair-capable strains compared with sensitive strains, which can be incorporated in genomic predictions of spore inactivation rates.

## Supplementary Material

Supplemental data
