# Supplementary material for: Predicting Inactivation of Bacillus subtilis Spores Exposed to Broadband and Solar Ultraviolet Light
Source: Environ Eng Sci. 2019 Jun 4;36(6):667–80. doi: 10.1089/ees.2018.0404 (PMC6588126; doi:10.1089/ees.2018.0404)
Supplement: Supplemental data [file Supp_Data.pdf]

Supplementary Data

Mathematical Symbols, Parameters, and Variables Used

a_i = Relative contribution of each damage mechanism to a summed Gaussian expression of the total probability of damage $p_{\text{total}}(\lambda) = \sum_{\text{damage types } i} a_i p_i(\lambda)$

A = Constant term in exponential model fit to survival data Ae^{-kF}

$\alpha(\lambda)$ = Dimensionless quantity, a function of wavelength λ, defined by $\alpha \equiv k_j(\lambda)F_{\text{ph}}(\lambda)$

$B(x, N, p)$ = Binomial probability mass function for x events occurring from N trials with each trial having probability p

c = Speed of light

$\mathbb{C}_{L,n}(k,j)$ = Array of n-combinations L objects with repetitions. k, j are indices of the array. Elements of the array are denoted $c_{i,j}$. Rows of the array are denoted $\mathbb{C}_{L,n\ \text{row}}$

E_i = Energy (eV) of peak probability in a Gaussian probability distribution (the mean) for probability p_i, where p_i is the damage probability for each damage type $i = 1, 2, 3$

IAS = Inactivation action spectrum, decay constant, or damage probability as a function of wavelength

$k_j(\lambda)$ = Exponential the decay constant $k_j(\lambda)$, a function of wavelength with subscript j indication units are m^2/J

k_s = Exponential decay constant for sensitive strain Bs, units m^2/J

k_{wt} = Exponential decay constant for the wild-type repair-capable strain Bs, units m^2/J

$\mathcal{K}$ = Polychromatic decay constant used in reference C2002 defined by $\mathcal{K} = \frac{1}{\mathcal{F}_0}\int_{200\text{nm}}^{400\text{nm}} k_w(\lambda)F(\lambda)d\lambda$, averaged over wavelength and weighted by fluence as indicated

λ = Wavelength of irradiation, typically in nm

F = Fluence in J/m^2, frequently used as a function of wavelength λ as in $F(\lambda)$

$F_{\text{ph}}(\lambda)$ = Fluence (in J/m^2) corresponding to 1 photon of wavelength λ per nm^2

$\mathcal{F}_0$ = Total polychromatic fluence as used in reference C2002, $\mathcal{F}_0 = \int_{200\text{nm}}^{400\text{nm}} F(\lambda)d\lambda$

h = Planck's constant

$I(\lambda)$ = Radiant intensity in W/(m^2-min), as a function of wavelength and exposure time t in minutes. If constant in time over the exposure $I \times t = F$, the fluence

L = The number of intervals in wavelength at which the total range of irradiation is divided to do calculations

$\mathcal{M}$ = The number of combinations of L items taken n at a time, with repetitions, $\mathcal{M} = \binom{L+n-1}{n}$

n = Shoulder parameter in multitarget survival function $S(F) = 1 - \left(1 - e^{-k_{wt}F}\right)^n$ typically varies with wavelength as $n(\lambda)$

$\frac{N(F)}{N_0}$ = Number of viable spores, $N(F)$ after exposure to fluence F divided by N_0, the number of viable spores present before exposure

$\mathcal{N}$ = Fluence weighted average over wavelength shoulder parameter for a multitarget survival function used by reference C2002 for polychromatic irradiation defined by $\mathcal{N} = \frac{1}{\mathcal{K}\mathcal{F}_0}\int_{200\text{nm}}^{400\text{nm}} n(\lambda)k_w(\lambda)\mathcal{F}(\lambda)d\lambda$

$p(\lambda)$ = The probability of damage (dimer lesion) to the DNA from an incident photon of wavelength λ

$p(\text{ss})$ = The probability of damage (dimer lesion) to the DNA from an incident photon for the sensitive strain (ss)

$p(\text{wt})$ = The probability of damage (dimer lesion) to the DNA from an incident photon for the wild-type (wt) repair-capable strain

p_i = The probability that a photon in wavelength interval centered on λ_i, where $i = 1, 2, 3\ldots$ will be absorbed and result in a damage site (dimer lesion)

p_1, p_2, p_3 = Probability of an incident photon being absorbed and forming a dimer lesion, dependent on whether previous damage has been incurred. p_1 is the probability if no previous damage has occurred, p_2 is the probability if one previous damage has occurred, p_3 is the probability if two previous damage events have occurred. When used with an explicit subscript as here refers to the conditional probability as described here, to be distinguished from the use of p_i, described above, where the index refers to wavelength intervals

$P(\mathcal{F})$ = Survival probability, given polychromatic fluence $P(\mathcal{F})$ as used in reference C2002, of the form $P(\mathcal{F}) = 1 - \left(1 - e^{-\mathcal{K}\mathcal{F}_0}\right)^{\mathcal{N}}$

$P(i;N)$ = The probability of i damage lesions occurring from N incident photons, used for the conditional binomial probability distribution

$P(k; n_{\text{ph}}, p)$ = Binomial probability distribution where the probability of exactly k damaged sites given n_{ph} incident photons is $P(k; n_{\text{ph}}, p) = \binom{n_{\text{ph}}}{k} p^k q^{n_{\text{ph}} - k}$ with p the probability of damage due to an incident photon and $q = 1 - p$ is the probability of no damage

$P_j = P_j = P(n$ damage events due j throw$)$ is the probability that n damage events occur from the event combination indexed by the jth row of $\mathbb{C}_{L,n}\ (k, j)$

P_{ND} = Probability that an incident photon results in no damage to the DNA; that is, the probability of survival of the spore

$q(\lambda)$ = Probability of all sites within the reference cross-sectional area surviving (not absorbing so as to form a dimer lesion) given an incident photon

q_i = The probability that a photon in wavelength interval centered on λ_i, where $i = 1, 2, 3\ldots$ will *not* be absorbed and result in a damage site (dimer lesion)

q_1, q_2, q_3 = Probability of an incident photon not being absorbed and forming a dimer lesion, dependent on whether previous damage has been incurred. q_1 is the probability if no previous damage has occurred, q_2 is the probability if one previous damage has occurred, q_3 is the probability if two previous damage events have occurred

$S(F)$ = Used to denote surviving fraction of spores as a result of fluence F

SID = Spore inactivation dose defined by $\mathrm{SID} = -\log\left(N_e/N_c\right)$, where N_e is the number of surviving spores after exposure to some fluence and N_c is the number of viable spores before exposure

SIDR = SID rate with $\mathrm{SID} = \mathrm{SIDR} \times t$, where surviving spores as a function of time are given by $S(t) = e^{-\int k_s(\lambda) I(\lambda) t d\lambda} \equiv e^{-\mathrm{SIDR} \times t}$

σ_i = Standard deviation of a Gaussian distribution for damage probability p_i, where p_i is the damage probability for each damage type $i = 1, 2, 3$

x_i = Number of damaged sites induced in wavelength intervals indexed by $i = 1, 2, 3\ldots$. Used when summing over the probabilities of damage to levels x_i in wavelength interval λ_i as in a cumulative binomial distribution, such as Equation (10)
